# Supplementary material for: Enabling high-temperature processing of thin film Li-ion batteries using a LISICON based solid-state electrolyte
Source: J Mater Chem A Mater. 2025 Dec 2;14(5):2989–97. doi: 10.1039/d5ta07144e (PMC12686852; doi:10.1039/d5ta07144e)
Supplement: TA-014-D5TA07144E-s001 [file TA-014-D5TA07144E-s001.pdf]

Supplementary Information (SI) for Journal of Materials Chemistry A.
This journal is © The Royal Society of Chemistry 2025

Supporting Information

Enabling high-temperature processing of thin film Li-ion batteries using a LISICON based solid-state electrolyte

Mohammadhossein Montazerian[1,2], Kyle J. Stephens[1,2], Nick A. Shepelin[*,1], Vladimir Roddatis[3], Christof Vockenhuber[4], Arnold Müller[4], Anders J. Barlow[5], Thomas Lippert[1,2], Daniele Pergolesi[*,1,6]

[1] Paul Scherrer Institute PSI, Center for Neutron and Muon Sciences, 5232 Villigen, Switzerland

[2] ETH Zürich, Department of Chemistry and Applied Biosciences, 8093 Zürich, Switzerland

[3] GFZ Helmholtz Centre for Geosciences, Telegrafenberg, 14473 Potsdam, Germany

[4] ETH Zürich, Department of Physics, 8093 Zürich, Switzerland

[5] Materials Characterisation and Fabrication Platform (MCFP), University of Melbourne, 3010 Victoria, Australia

[6] Paul Scherrer Institute PSI, Center for Energy and Environmental Science, 5232 Villigen, Switzerland

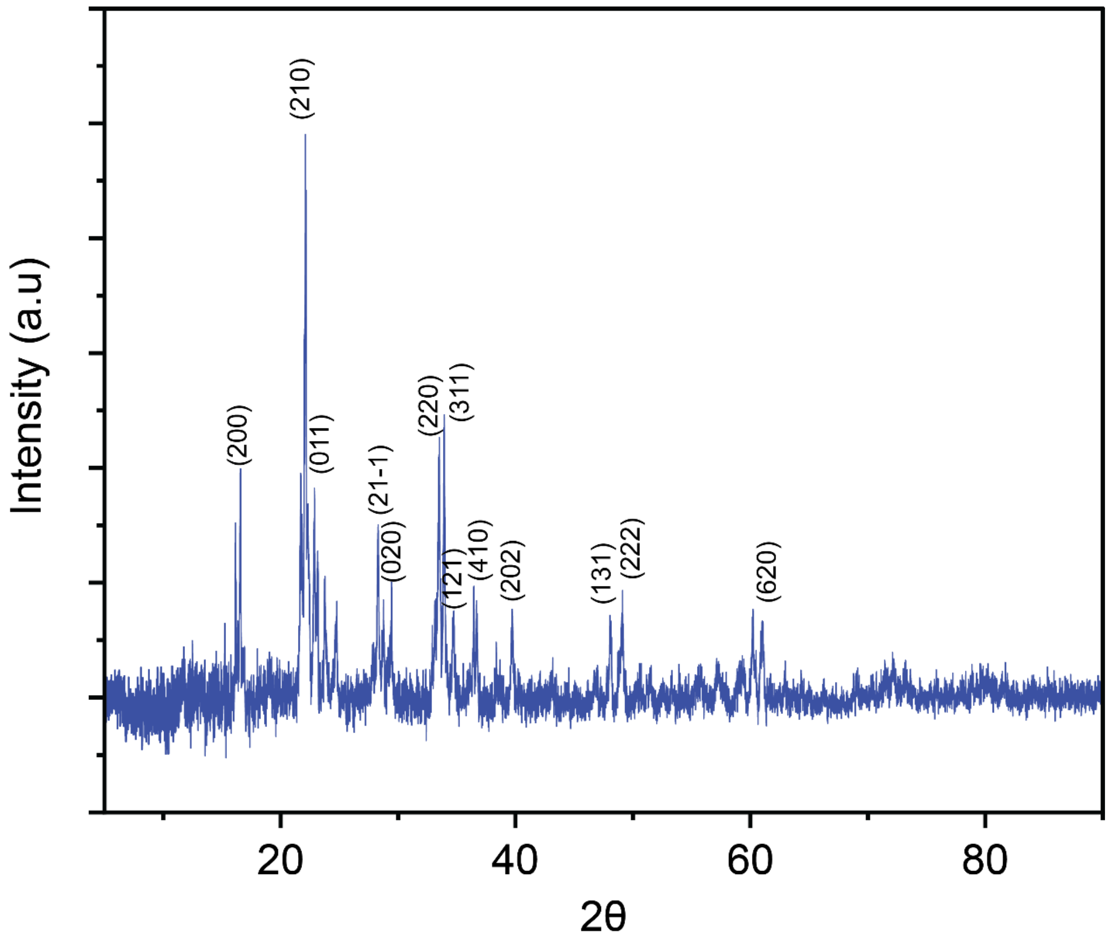

Figure S1. X-Ray diffractogram of LGPO PLD target's powder.

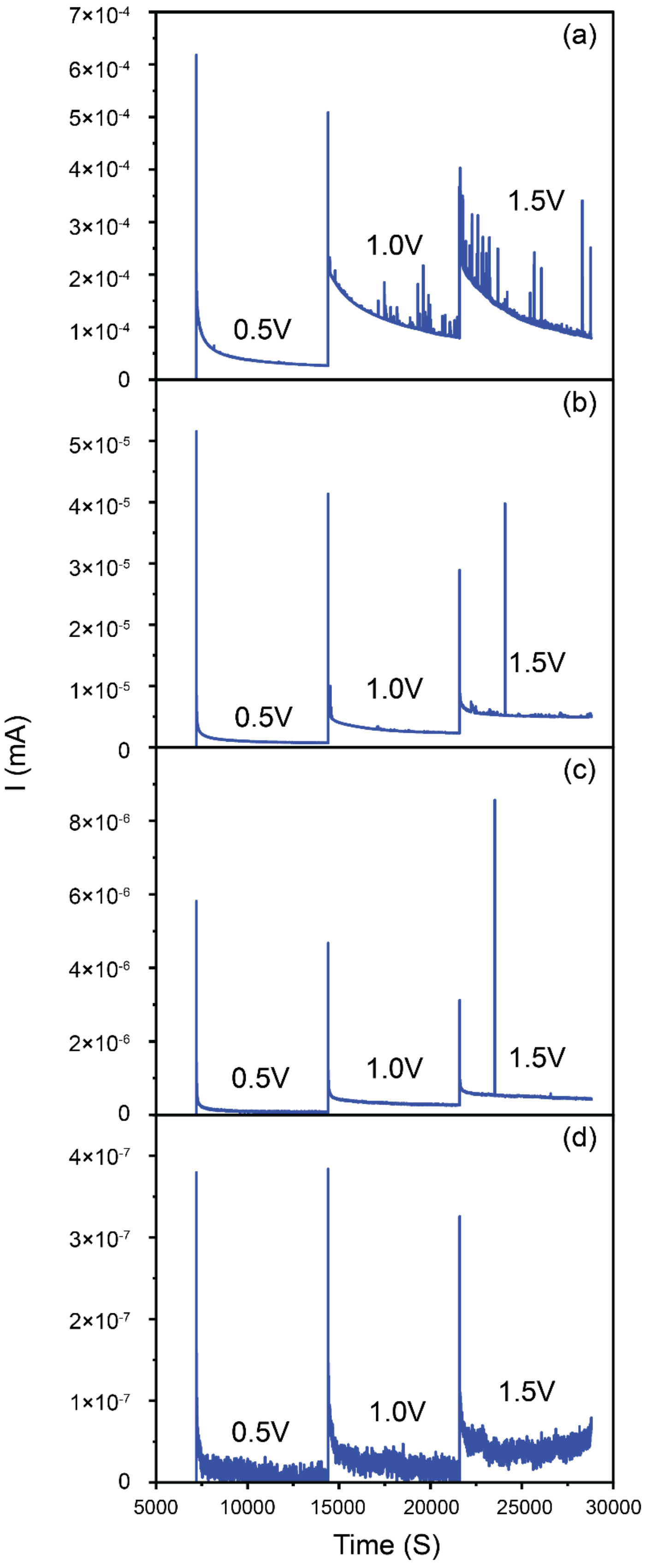

Figure S2. The current-time plots of the HTLP sample at (a) 535˚C, (b) 450˚C, (c) 370˚C, (d) 290˚C.
